# Supplementary material for: Stacking Model‐Based Classifiers for Dealing With Multiple Sets of Noisy Labels
Source: Biom J. 2025 Mar 12;67(2):e70042. doi: 10.1002/bimj.70042 (PMC11898607; doi:10.1002/bimj.70042)
Supplement: Supplementary file 1 — Supporting Information [file BIMJ-67-e70042-s001.zip › density-based-ensemble-model-main/competitors_real_data/netlab-master/netlab-master/help/gmminit.htm]

Netlab Reference Manual gmminit


# gmminit

## Purpose

Initialises Gaussian mixture model from data

## Synopsis

```
mix = gmminit(mix, x, options)
```

## Description

`mix = gmminit(mix, x, options)` uses a dataset `x`
to initialise the parameters of a Gaussian mixture
model defined by the data structure `mix`. The k-means algorithm
is used to determine the centres. The priors are computed from the
proportion of examples belonging to each cluster.
The covariance matrices are calculated as the sample covariance of the
points associated with (i.e. closest to) the corresponding centres.
For a mixture of PPCA model, the PPCA decomposition is calculated
for the points closest to a given centre.
This initialisation can be used as the starting point for training the
model using the EM algorithm.

## Example

```
mix = gmm(3, 2);
options = foptions;
options(14) = 5;
mix = gmminit(mix, data, options);
```

This code sets up a Gaussian mixture model with 3 centres in 2 dimensions, and
then initialises the parameters from the data set `data` with 5 iterations
of the k means algorithm.

## See Also

`gmm`

---

**Pages:**
Index


---

Copyright (c) Ian T Nabney (1996-9)
